# Expression of mTOR in normal and pathological conditions

**DOI:** 10.1186/s12943-023-01820-z

**Published:** 2023-07-15

**Authors:** A Marques-Ramos, R Cervantes

**Affiliations:** 1 H&TRC-Health & Technology Research Center, ESTeSL-Escola Superior de Tecnologia da Saúde, Instituto Politécnico de Lisboa, Lisbon, Portugal; 2 Public Health Research Centre, NOVA National School of Public Health, Universidade Nova de Lisboa, Lisbon, Portugal; 3 Comprehensive Health Research Center (CHRC), Lisbon, Portugal

**Keywords:** mTOR expression, mTOR transcriptional regulation, mTOR translation regulation, mTOR mRNA stability, mTOR expression disease, mTOR expression cancer, mTOR biomarker

## Abstract

The mechanistic/mammalian target of rapamycin (mTOR), a protein discovered in 1991, integrates a complex pathway with a key role in maintaining cellular homeostasis. By comprising two functionally distinct complexes, mTOR complex 1 (mTORC1) and mTORC2, it is a central cellular hub that integrates intra- and extracellular signals of energy, nutrient, and hormone availability, modulating the molecular responses to acquire a homeostatic state through the regulation of anabolic and catabolic processes. Accordingly, dysregulation of mTOR pathway has been implicated in a variety of human diseases. While major advances have been made regarding the regulators and effectors of mTOR signaling pathway, insights into the regulation of mTOR gene expression are beginning to emerge. Here, we present the current available data regarding the mTOR expression regulation at the level of transcription, translation and mRNA stability and systematize the current knowledge about the fluctuations of mTOR expression observed in several diseases, both cancerous and non-cancerous. In addition, we discuss whether mTOR expression changes can be used as a biomarker for diagnosis, disease progression, prognosis and/or response to therapeutics. We believe that our study will contribute for the implementation of new disease biomarkers based on mTOR as it gives an exhaustive perspective about the regulation of mTOR gene expression in both normal and pathological conditions.

## Background

The mechanistic target of rapamycin (mTOR) is a serine/threonine kinase that coordinates metabolism and growth of eukaryotic cells with external inputs such as nutrition and growth stimuli [1]. Over the last two decades, extensive research has demonstrated that mTOR is involved in key cellular processes, from protein synthesis to autophagy, and that hyperactivated mTOR signaling has been linked to cancer, diabetes, and the aging process [2]. It is a key component of two complexes, mTOR complex 1 (mTORC1) and mTORC2 that display several functions according to different downstream effectors [2]. mTORC1 exerts its effects through phosphorylation of several proteins, particularly 4E-binding proteins (4E-BPs) and S6 kinases (S6Ks), which induces protein synthesis, lipid and nucleotide biogenesis, and suppresses autophagy, lysosomal biogenesis, ultimately resulting in cell survival, growth and proliferation [2, 3]. mTORC2 targets several protein kinases, including Akt, by which it induces cell survival and proliferation [4]. Dysregulation of mTOR is present in a myriad of diseases and it has been reported that mTOR hyperactivation occurs in more than 70% of human cancers [4]. Accordingly, the regulators of mTOR pathway have been the subject of several studies that, recently, expanded to the understanding of how mTOR expression itself is regulated. Furthermore, the investment in the development of biomarkers has been exponential and, in this scope, several authors are addressing the expression of mTOR in different pathological conditions, such as Type 2 Diabetes Mellitus [5, 6], Alzheimer [7], rheumatoid arthritis [8] and in several types of cancers [9, 10]. Accordingly, this review aims to systematize the current knowledge about the regulation of mTOR expression and to address the potential of mTOR as a biomarker for diagnosis, prognosis and/or therapeutic response.

## Overview of mTOR signaling

**Fig. 1:**
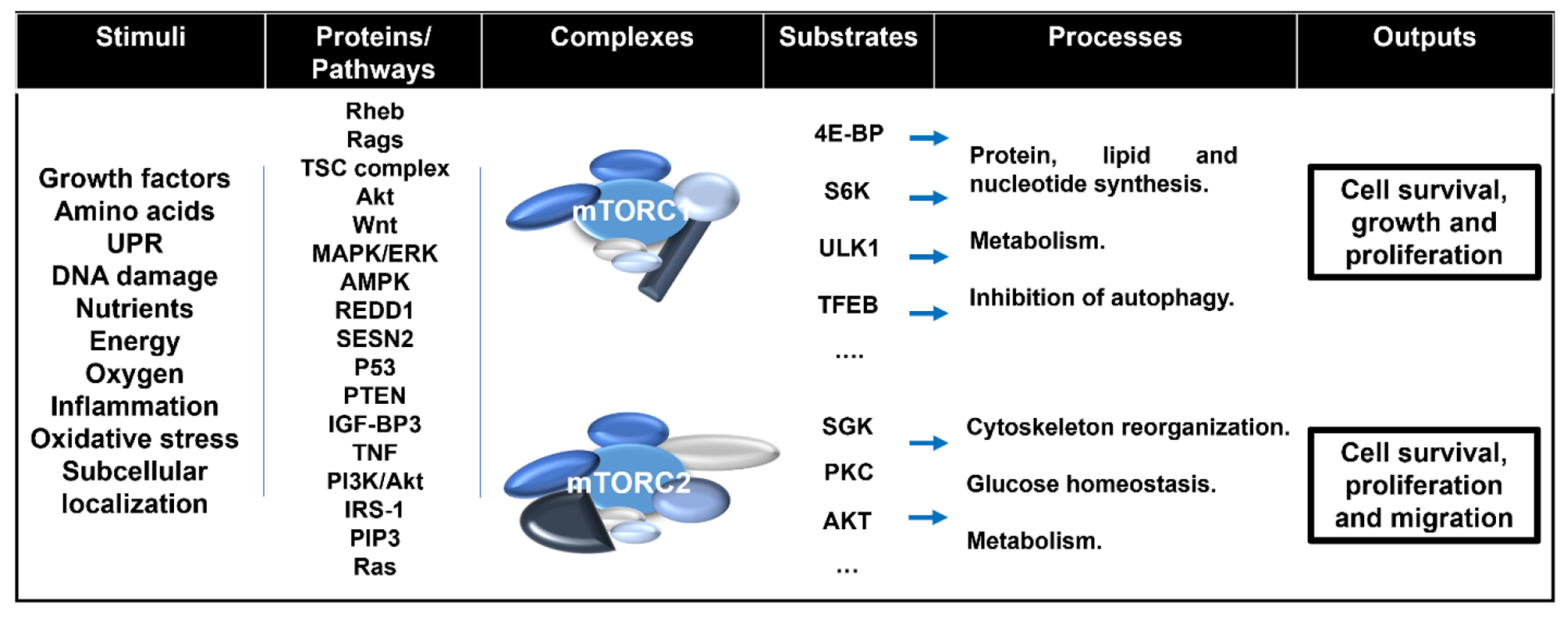
Signals, pathways, targets, and outputs of the mTOR signalling. mTOR is a protein kinase that complexes with several proteins composing the mTOR complex 1 (mTORC1) and mTORC2. Both are activated by growth factors and mTORC1 also depends on amino acids for its translocation into the lysosome membrane where it becomes fully activated. These processes involve the activation of the Rag GTPases Rag A or Rag B and Rag C or D complexed with Ragulator complex in addition to the Ras homolog enriched in brain (RHEB). Additional signals that result in mTORC1 activation include insulin and inflammation, that act through the insulin-like growth factor-1 (IGF-1)/AKT and the TNF/Tuberous Sclerosis Complex (TSC) axis, respectively. On the other hand, inactivation of mTORC1 occurs under stress conditions, such as energy starvation, a process dependent on AMPK; hypoxia, through upregulation of REDD1, endoplasmatic reticulum stress (UPR) by upregulation of Sestrin 2 (SESN2); and DNA damage, by activation of the p53 transcriptional program. mTORC2 becomes activated in the plasma and mitochondrial membranes, a subpopulation of endosomal vesicles and in the nucleus, through site-specific processes. Each complex has a plethora of substrates, such as but not exclusively 4E-binding proteins (4E-BP), S6 kinases (S6K), unc-51-like kinase (ULK1) and Transcription factor EB (TFEB) for mTORC1 and glucocorticoid-induced kinases (SGK), protein kinase C (PKC) and AKT for mTORC2. The main biological processes regulated by mTORC1 include protein, lipid and nucleotides synthesis, metabolism and autophagy; and cytoskeleton reorganization, glucose homeostasis and metabolism for mTORC2, whose activation results in cell survival, growth, proliferation and migration

The research of TOR began in 1960s with a journey to Rapa Nui (also known as Easter Island), to discover natural compounds, from plants and soil, with potential medicinal use. There, a natural macrolide was identified and in 1972 Suren Sehgal isolated it from a bacteria called *Streptomyces hygroscopicus*, refined it, and reported it to have powerful anti-fungal action. This compound was called *Rapamycin* in honor of its source and action [11]. Although rapamycin was first identified as an antifungal metabolite, it was later proven to have immunosuppressive and anti-proliferative characteristics in mammalian cells, motivating researchers’ interest in understanding how it operates [12]. In particular, in 1981, the National Cancer Institute tested rapamycin in about 60 tumour cell lines and found that this compound inhibited growth of cells from a variety of origins, such as mammary and colon cancers, melanocarcinoma, and ependymoblastoma, which pointed rapamycin as a priority drug [13]. In the following years, the anti-tumoral potential of rapamycin as a growth inhibitory molecule was extended to organisms such as *Saccharomyces cerevisiae* [14], *Drosophila* [15, 16], *Caenorhabditis elegans* [17], fungus [18], plants [19], and mammals [20]. Along with these discoveries, several attempts were undertaken to study the cellular effects of this compound, in particular its targets. As such, in 1991, Michael Hall and Joseph Heitman identified the protein *target of rapamycin* (TOR) as the cellular target of rapamycin in *Saccharomyces cerevisiae* [14], and, three years later, four laboratories independently identified the mammalian orthologue of TOR, now recognized as mechanistic target of rapamycin (mTOR) [21–24]. First, mTOR was linked to the regulation of cell cycle and proliferation [25]. Now it is known that this serine/threonine kinase belongs to the phosphatidylinositol 3-kinase-related kinase (PIKK) family and that it displays a plethora of functions through the regulation of cell development and metabolism in response to environmental cues, ensuring that cells expand only under favourable conditions [25] (Fig. 1). When activated, mTOR signalling promotes cell growth and proliferation by stimulating biosynthetic pathways such as protein, lipid, and nucleotide production and by inhibiting cellular catabolism via inhibition of the autophagy pathway [26]. According to its biochemical and genetic analysis, it is known that in eukaryotic cells mTOR is found in two functionally different complexes, mTORC1 and mTORC2, that target, by phosphorylation, distinct substrates, contributing to diverse physiological roles [20]. These complexes also have different sensitivity to rapamycin, as while mTORC1 is susceptible to this macrolide, mTORC2 is resistant to acute exposure but not to prolonged treatment [27]. mTORC1’s main components are mTOR, mammalian lethal with sec-13 protein 8 (mLST8), and the TOR regulatory associated protein (RAPTOR). Additional components are the DEP-domain containing mTOR interacting protein (DEPTOR) and Proline-rich Akt substrate 40 kDa (PRAS40) [27]. The core of mTORC2 is composed by mTOR, mLST8, rapamycin-insensitive companion of mTOR (RICTOR), stress-activated protein kinase-interacting protein 1 (mSIN1). Additional regulatory components are PROCTOR 1/2 and DEPTOR [2, 27]. These complexes are also activated differently, as mTORC1 is activated in the lysosome by both amino acids and growth factors, whereas mTORC2 is primarily induced by growth factors and is activate in different cellular compartments [1].

The major regulators of mTORC1 are Rag GTPases and the Ras homolog enriched in brain (RHEB). When the environment is appropriate for cell growth, the Rag heterodimer recruits mTORC1, through binding to RAPTOR, from the cytoplasm to the lysosome membrane where it will co-localize with RHEB. When activated, GTP-bound RHEB binds and stimulates mTORC1 [28–31]. The Rag heterodimers are composed by Rag A or B and Rag C or D and, by association with the Ragulator complex, are found at the lysosomal membrane [32]. The activation of Rag heterodimers require the presence of amino acids, that inhibit CASTOR1 which, ultimately, relieves the inhibition exerted by GATOR1 in the Rag heterodimers [1]. In turn, RHEB is activated by growth factors, that activate AKT, which inhibits the tuberous sclerosis complex (TSC) trough phosphorylation of its component TSC2. The phosphorylation of TSC2 prevents it from associating with TSC1 to form, along with TBC1D7, the functioning complex [33, 34]. The inhibition of the TSC complex results in RHEB activation as this complex is a GTPase-activating protein (GAP) of RHEB [33, 34]. Accordingly, as mTORC1 requires both Rags and RHEB, its activation only occurs in the presence of both **amino acids** and **growth factors**.

The TSC complex is a hub of external and internal stimuli, modulating the activity of mTORC1. It is inhibited by **insulin** through insulin-like growth factor-1 (IGF-1)-mediated activation of AKT, resulting in mTORC1 activation, as explained earlier. **Growth factors** also modulate mTORC1 activity through the activation of the **Wnt and MAPK/ERK pathways**, that inhibit TSC through phosphorylation by GSK3 or ERK and RSK, respectively [35–37]. In addition, upon growth factor stimulation, AKT directly phosphorylate and inactivate PRAS40, a mTORC1 inhibitor [38, 39]. mTORC1 also integrates signals from **cellular energy** through AMPK that, under energy starvation, phosphorylate both the TSC complex [40], and RAPTOR [41], which, in this case, results in mTORC1 inhibition [40, 41]. Linking **inflammation** with mTORC1 signaling, is the fact that TNF also inhibits TSC with concomitant mTORC1 activation [42]. On the other hand, **hypoxia** activates TSC through upregulation of REDD1, which results in mTORC1 inhibition [43, 44]. Similarly, in **endoplasmic reticulum stress** mTORC1 is inhibited, although in a TSC-independent fashion, by the increase of Sestrin 2 (SESN2) expression, an inhibitor of the Rag-Ragulator complex [45]. Upon **DNA damage**, the p53 transcriptional program is activated, of which the increase of AMPKβ1, AMPKβ2, TSC, PTEN and IGF-BP3 result in inhibition of mTORC1 [46]. As a part of a negative feedback, the TSC complex is inactivated by the mTORC1 substrate S6K1, that will reduce the expression or phosphorylation of the insulin receptor substrate 1 (IRS-1) [47, 48].

As for mTORC2, it is regulated primarily by **growth factors**, and its activity is observed in the plasma membrane, mitochondrial membranes, a subpopulation of endosomal vesicles and in the nucleus [49–51].The mTORC2 complex is regulated by the IRS/PI3K axis, in which the growth factor-derived phosphatidylinositol [3–5]-trisphosphate (PIP3) relieves the inhibitory role of mSIN1 on mTORC2 [52]. In addition, the Ras pathway is now known to regulate mTORC2 activity, not only indirectly through PI3K, but also via direct activation [53–56]. Besides growth factors, mTORC2 is also sensitive to **nutrients**, although to a different extent than mTORC1. For short, an increase in mTORC2 activity is observed upon deprivation of nutrients, particularly glutamine and glucose [57, 58]. In addition, it has been demonstrated that the **subcellular localization** of mTORC2 also plays a key role in regulating its activity [59]. The regulation at each location is now being unrevealed, but it is known that the activation of mTORC2 at the plasma membrane and mitochondria-associated ER membrane (MAM) requires growth factor stimulation, as explained earlier, in opposition to mitochondria in which mTORC2 is activated in a PI3K-independent fashion [49]. Similarly, mTORC2 associates with translating **ribosomes** in an insulin-dependent manner, and this association seems to be required for mTORC2 activation [60, 61]. Other routes that lead to mTORC2 activation include AMPK, through phosphorylation of both mTOR and RICTOR [62]; the canonical Wnt pathway [63, 64]; the Hippo pathway [65]; TGF-β [66, 67] and GSK-3 [68].

These two complexes also regulate each other, in a way that mTORC2 activates mTORC1, whereas mTORC1 blocks mTORC2. One way by which mTORC2 activates mTORC1 is through phosphorylation and activation of AKT [4]. In turn, mTORC1 inactivates IRS-1 in a S6K1-dependent manner, resulting in PI3K pathway inhibition [47, 48] and induces GRB10 that inhibits IRS-1 and, thus, the PI3K/insulin signalling [69, 70].

Most of the aforementioned signals converge on mTOR signalling, particularly mTORC1, through phosphorylation of mTOR protein itself, that can occur at multiple locations. The majority of the phosphorylation events result in mTORC1 activation (Thr 2446 and Ser 2448, Ser 2448 alone, Ser 1261, Ser 2481, Ser 1415, Ser 2159 and Thr 2164), nevertheless a negative regulation has also been observed upon mTOR phosphorylation (Thr 2446 alone) [71].

## Methodology

This review was performed using the PICO methodology, where P represents the Studies assessing mTOR expression regulation or changes in mTOR expression in cellular stress/pathological conditions; I is whether mTOR expression is regulated in normal, cellular stress and/or pathological conditions; C represents normal cellular conditions; and O is whether mTOR expression is specifically regulated at the translational level and is increased in stress/pathological conditions.

The study reports available data published from 1 to 2006, in English. Inclusion criteria were articles published from January 2006, which characterize regulation of mTOR expression in normal and pathological conditions. Articles that didn’t fulfil the inclusion criteria were not subjected to additional review, namely, review articles, non-English language articles, and articles that address the signaling pathway but not mTOR expression.

The selection of the articles was performed through Zotero in three rounds. The first round included a screening of all titles to exclude papers that were duplicated or unrelated to the topic. The second round consisted of an abstract screening. In the third round, considering the inclusion and exclusion criteria, the entire texts of all possibly relevant papers were evaluated. The following data was manually compiled: [1] Title, [2] The effect of mTOR, [3] Pathology, [4] Methods, [5] Main Findings, and [6] References. We included 59 studies where 12 was regarding regulation of mTOR expression and 47 were studies regarding mTOR expression levels and disease.

### Regulation of mTOR expression

Since the discovery of mTOR, a plethora of groups dedicated their efforts in understanding the mTOR pathway. As result, in about 30 years of research over 45 000 studies were published addressing mTOR and the mTOR signaling pathway. The overwhelming majority of these studies address the regulators and/or effectors of both mTORC1 and mTORC2 pathways. Nevertheless, recently, some studies have been elucidating the regulation of mTOR expression itself in terms of transcription, translation, and mRNA stability. It is also known that mTOR and the mTOR signaling pathway are regulated by miRNAs and other non-coding mRNAs, a topic that will be addressed elsewhere.

#### mTOR transcription regulation

**Fig. 2:**
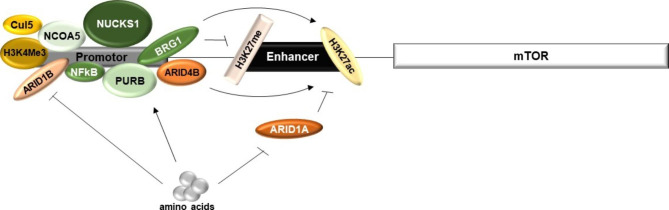
Regulation of mTOR transcription. Amino acids increase mTOR transcription through induction of binding of transcriptional activators such as Nuclear Receptor Co-Activator 5 (NCOA5), Purine-Rich Element Binding Protein B (PURB), cyclin-dependent kinase substrate 1 (NUCKS1), and nuclear factor of kB (NFkB) to the *mTOR* promoter. In addition, amino acids induce the degradation of AT-rich interaction domain 1 A (ARID1A) and ARID1B, which result in increased *mTOR* transcription, through relieve of the inhibitory effect of H3K27ac, an epigenetic modified histone that marks for active enhancers; and the reversal of the inhibitory effect of ARID1B on *mTOR* promoter, respectively. The binding of H3K27ac to *mTOR* is further regulated by ARID4B and Brahma-related gene 1 (BRG1), that bind themselves to the promoter of *mTOR* in an amino acid-dependent manner. BRG1 additionally relieves the inhibitory effect of H3K27me3 on *mTOR* transcription. Other inducers of *mTOR* transcription upon amino acid stimulation, particularly, taurine, include the epigenetic marker for promoter activation H3K4Me3 and Cullin 5 (Cul5), an ubiquitin ligase that is highly expressed in mammary gland tissues in the lactation stage

The regulation of transcription is a key event in the regulation of gene expression. It can assume a variety of forms, such as epigenetic mechanisms, assembly of the transcriptional apparatus or the process of transcription itself: at the initiation, elongation or termination phases [72]. For mTOR, by studying the process of milk synthesis in mammary cells, it has been demonstrated that the presence of amino acids potentiates mTOR transcription through binding of the transcriptional activators Nuclear Receptor Co-Activator 5 (NCOA5), Purine-Rich Element Binding Protein B (PURB), cyclin-dependent kinase substrate 1 (NUCKS1), and nuclear factor of kB (NFkB) to the *mTOR* promoter [73–76] (Fig. 2). Additionally, in these settings, the presence of amino acids induces the degradation of AT-rich interaction domain 1 A (ARID1A) and ARID1B, which resulted in increased *mTOR* transcription [77, 78]. ARID1A is an inhibitor of H3K27ac, an epigenetic modified histone that marks for active enhancers [79]. Accordingly, the TRIM21-mediated ubiquitination and proteasomal degradation of ARID1A results in increased *mTOR* transcription [77]. Similarly, the Brahma-related gene 1 (BRG1), another component of the mammalian switch/sucrose non-fermentable chromatin remodelling complex, which expression and binding to *mTOR* promoter are stimulated by isoleucine, induces the binding of H3K27ac to the mTOR promoter whereas has the opposite effect for H3K27me3 [80]. The result is the induction of *mTOR* transcription, as the deposition of H3K27me3 on gene enhancers is an epigenetic mark of gene inhibition [81]. Furthermore, it was found that ARID4B, another protein that regulates the binding of H3K27ac to the *mTOR* promoter, also associates with *mTOR* promoter itself and that this binding is stimulated by Taurine (Tau) [82]. Nevertheless, ARID4B association with *mTOR* potentiates rather than inhibits H3K27ac, which results in increased *mTOR* transcription [82]. In addition, Tau also stimulates the binding of the trimethylation histone H3 lysine 4 (H3K4Me3) to the *mTOR* promoter [83]. H3K4Me3 is an epigenetic marker for promoter activation [84]. It seems that the increase of *mTOR* mRNA levels induced by Tau is mediated by Cullin 5 (Cul5), an ubiquitin ligase that is highly expressed in mammary gland tissues in the lactation stage [85]. The mechanism by which Cul5 regulates *mTOR* mRNA levels remains unknown [85]. Similarly, it was found that the protein Brahma (BRM), a chromatin remodelling and histone modification factor, also binds to the *mTOR* promoter, particularly in the presence of leucine, and that this binding results in induction of *mTOR* transcription [86]. Curiously, ARID1A, BRG1, BRM, ARID4B, H3K27ac and H3K27me3 all share the same binding site at the *mTOR* promoter [77, 80, 82, 86], suggesting the presence of a cis-acting element, such as an enhancer. In addition, in these settings, it was observed that the association of these transcriptional activators not only increased *mTOR* transcript levels, but also induced mTOR phosphorylation and, thus, mTOR signalling activation. Nevertheless, the increase of *mTOR* mRNA levels did not impact the levels of unphosphorylated mTOR. It remains to be determined whether a post-transcriptional event is also regulating mTOR expression during milk synthesis.

#### mTOR translation regulation

**Fig. 3:**
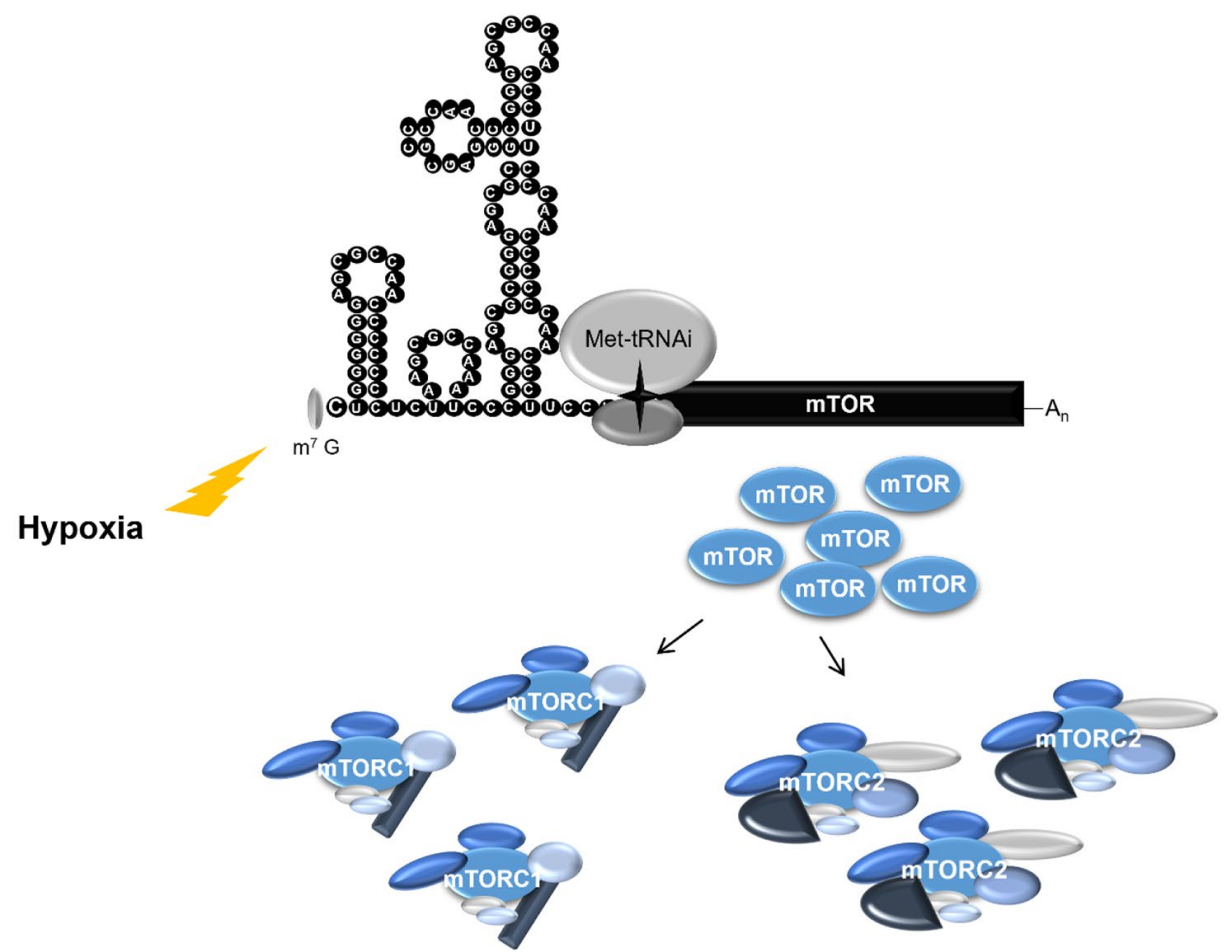
mTOR is translated in a cap-independent manner. mTOR 5’UTR adopts a highly folded and evolutionary conserved structure, that is capable to directly bind to the 40 S ribosomal subunit in the absence of any initiation factor. This RNA scaffold assists cap-independent translation of mTOR, allowing sustained mTOR protein levels in translational inhibitory conditions (hypoxia). Cap-independent translation of mTOR occurs both in normal and stress conditions and is necessary for mTOR function

It is widely known that mTOR signalling operates and is necessary in a variety of physiological conditions associated with global protein synthesis reduction, such as in hypoxia and mitosis [87, 88] and that the mTOR protein levels itself remain unchanged in those settings [87]. Furthermore, it has been observed that some pathological conditions such as systemic lupus erythematosus are associated with a reduction in *mTOR* mRNA levels but an increase in mTOR protein levels [89] .These data suggest that mTOR is subjected to regulation at the translational level. Indeed, our group demonstrated that mTOR is translated by an alternative and cap-independent mechanism that operates both in normal and stress conditions, allowing sustained mTOR protein levels regardless of the translational inhibitory cues [90] (Fig. 3). These findings might explain how mTOR is capable to be activated in a variety of physiological settings strongly associated with protein synthesis reduction. Furthermore, it gives a cue of how mTOR evades the normal translational checkpoints and is over-expressed in a variety of diseases, as discussed below, as its translation is independent of cap and the initiation factors that are usually blocked by the control mechanisms of the cell [90]. Additionally, it opens a new avenue to counteract mTOR hyperactivation through reduction of mTOR expression, as our group is exploring.

#### mTOR mRNA stability regulation

The role of the RNA-binding protein La related protein 1 (LARP1) in the mTOR pathway has been decoded in the last years. It is a downstream effector of mTORC1, being phosphorylated by mTOR after binding to Raptor [69, 70], to control the translation of mRNAs with Terminal Oligopyrimidine (TOP) motifs [91–94]. Interestingly, it is now known that LARP1 also regulates mTOR in a post-transcriptional manner [95]. By studying the LARP1 interactome, Mura et al., 2015 demonstrated that LARP1 binds to *mTOR* mRNA, in the 3´UTR, and that this association promotes *mTOR* mRNA stability [95]. It remains to be determined how this binding occurs, whether it is through a cis-acting element in the *mTOR* mRNA 3’UTR and/or if it involves trans-binding of other proteins or mRNA elements.

N^6^-methyladenosine (m^6^A) is an mRNA modification consisting of the methylation at N^6^-position of adenosine at the RRACH sequence (where R = A or G, H = A, C, or U) [96]. This post-trancriptional modification results in regulation of gene expression as it has the potential to alter mRNA translation, degradation, splicing, export and/or folding [96]. It was demonstrated that, in the endometrium, m^6^A methylation induces the degradation of *mTOR* mRNA and other mTORC2 subunits, and that decreased m^6^A levels might contribute to the carcinogenesis of various cases of endometrial cancer [97]. In addition, in gastrointestinal cancer (GI), besides mTOR other members of the PI3K/Akt/mTOR pathway have very high confidence m^6^A modification sites and it has been demonstrated that this methylation event directly regulate PI3K/Akt/mTOR activation in GI cancer [98]. It seems to be determined whether m^6^A at mTOR mRNA is restricted to endometrial and gastrointestinal cancer and if mTOR is subjected to other RNA modifications.

### Changes in mTOR expression levels in diseases

Changes in the expression of specific genes is a signature of several diseases [99]. As for mTOR, the current literature demonstrates that its expression fluctuates in diseases such as brain, lung, skin, gastrointestinal and blood diseases (Table 1), as well as in several forms of cancer (Table 2).

**Table 1:** mTOR expression profile in pathological settings

Disease/Condition	mTOR Expression Level	Ref.
**BRAIN**		
Alzheimer	β-amyloid increased the expression of mTOR and p-mTOR (at Ser2448) and mTOR translocation to the nucleus	[100]
Vascular dementia	mTOR and p-mTOR protein levels were decreased at 21- and 28-days after chronic cerebral hypoperfusion (CCH) in the hippocampal CA1 region.	[101]
**LUNG**		
Idiopathic pulmonary fibrosis	The expression of mTOR correlated with the fibrosis score and lung function decline.	[102]
**SKIN**		
Dermatitis	mTOR expression was significantly increased in psoriasis, allergic contact dermatitis and atopic dermatitis.	[103]
Acne	mTOR expression was increased in the skin of acne patients (either in involved or non-involved skin)	[104]
Pathological scar	Increased mTOR expression in pathological scar fibroblasts.	[105]
**GASTROINTESTINAL TRACT**		
Diabetic nephropathy	Elevated mTOR mRNA levels	[106]
Lupus Erythematosus	Increase in mTOR protein levels in liver samples from a murine model of systemic lupus erythematosus, despite a reduction in mRNA levels.	[89]
**BLOOD**		
Blood	mTOR expression in peripheral blood of patients with osteoarthritic vary from high to low, in which high levels are associated with increased incidence of synovitis.	[107]
	In cord blood cells, the presence of IL2, IL7 and IL15 altogether resulted in an increase of mTOR expression at day 14, and a decrease at day 21. The reduction of mTOR expression was observed when cells were treated with IL2 or IL15 alone but not IL7.	[108]

#### Changes in mTOR expression in non-cancerous conditions

In **brain**, mTOR complex hyperactivation is known to be involved in several diseases [109], being a potential pharmacological target in conditions such tuberous sclerosis complex [110]. Regarding to its expression, a study addressing several types of tissues determined that, in normal settings, mTOR expression is elevated in the choroid plexus [111]. Furthermore, treatment of neurons with β-amyloid results in increased expression of mTOR, mTOR translocation into the nucleus and activation of mTOR signaling [100]. These data indicates that mTOR as a role in the etiology of Alzheimer’s disease [100]. On the other hand, reduction of mTOR protein and activation levels seem to play a role in vascular dementia as it is observed in the hippocampus of rat after chronic cerebral hypoperfusion (CCH) induced by permanent bilateral common carotid arteries occlusion (2VO) [101]. Interestingly, this decrease in both mTOR and p-mTOR protein levels, that was observed 21- to 28- after CCH, was preceded by a significant increase in p-mTOR levels at day 7 after treatment [101]. The authors were unable to determine the role of these fluctuations in p-mTOR expression upon CCH, but hypothesized that it might be related to the role of mTOR in cell survival [101]. Nevertheless, it would be interesting to test this hypothesis and further explore the role of mTOR upon CCH.

In **lungs**, by addressing biopsies of patients with idiopathic pulmonary fibrosis, Park and colleagues verified that increased mTOR expression was associated with high fibrosis score and lung function decline, indicating that high mTOR levels might be related to a poor prognosis of the disease [102]. In **skin** samples, elevated mTOR protein levels were observed in cutaneous inflammatory process, such as psoriasis, allergic contact dermatitis, atopic dermatitis, and acne [103, 104]. Similarly, elevated mTOR levels seems to be associated with the development of **glomerular diseases**, such as diabetic nephropathy, nevertheless, the authors also found that mTOR complex activity is necessary for correct podocyte homeostasis [106]. In **liver**, in which mTOR expression is usually low [112], increased mTOR protein levels were found in samples from a murine model of systemic lupus erythematosus and it was hypothesized that insulin stimulation was assisting this overexpression [89]. Of note, in these settings, it was discovered that mRNA levels were decreased, despite elevated protein levels, suggesting post-transcriptional regulation.

Accordingly, from the analyzed studies, it is apparent that fluctuations in mTOR expression occur at the onset and/or progression of some diseases and that increased protein levels are associated with deleterious effects and contribute to the etiology of some diseases or to a poor prognosis.

#### Changes in mTOR expression in cancer

Regarding to human cancers, it is evident that mTOR expression is also altered in a variety of tumoral specimens and that it might change throughout the course of the disease (Table 2).

**Table 2:** – mTOR expression profile in cancer

Cancer	mTOR Expression Level	Ref.
**Lung**	Meta-analysis demonstrated that there is no association between mTOR and p-mTOR expression and the prognosis of non-small cell lung cancer.	[113]
	In EGFR-mutant NSCLC samples mTOR expression was: low or intermediate in 62.5% of the cases and high 37.5%. The group with high mTOR and BIM expression had shorter overall and progression-free survival to erlotinib.	[114]
**Gastric**	Gastroenteropancreatic neuroendocrine tumours presented high levels of mTOR, 4EBP1, p-4EBP1, p-S6K and p-eIF4E. Both expression and activity of mTOR were higher in foregut than in midgut tumours. In foregut tumours, expression of mTOR was higher when distant metastases were present.	[115]
	Immunohistochemistry of paraffinembedded sections from gastric cancer cases reveled that mTOR expression was present in 51.5% (17/33) of the samples, in opposition to the low/absent expression in normal tissues. A positive correlation was observed between mTOR expression and tumor differentiation, lymph node metastasis and clinical staging. No correlation was observed with gender, age and invasive depth.	[116]
	Higher expression of mTOR and p-mTOR in the tumor center compared to the invasive front.	[117]
**Liver**	Expression of mTOR was elevated in patients with multinodular hepatocellular carcinoma and increased phospho-mTOR in tumoral tissue was associated with higher hepatocellular carcinoma recurrence rates after liver transplantation.	[118]
**Esophageal**	PI3K/Akt/mTOR signaling hyperactivation was accompanied with overexpression of mTOR. Expression of mTOR was elevated in tumor tissues in opposition to normal samples. High expression of mTOR and other mTOR signaling components were closely related to higher tumor size, lymph nodes metastases and advanced TNM stage. Overexpression of mTOR was proved to be an independent adverse prognostic factor for overall survival.	[119]
**Laryngeal carcinoma**	Expression of mTOR ranged from 0.0–80.2% and higher expression of mTOR was associated with increased recurrence and shorter disease-free survival.	[120]
	Expression of mTOR was higher in patients with disease recurrence and was associated with shorter disease-free survival.	[121]
**Urothelial carcinoma**	mTOR protein levels were elevated in tumours of urothelial patients that did not respond to neoadjuvant chemotherapy and decreased in complete responders.	[122]
**Pancreatic**	Expression of mTOR observed in about 71% of cases and correlated with p-mTOR expression. Patients with positive p-mTOR expression seemed to have shorter survival duration.	[123]
**Breast**	From the analysed luminal breast cancer specimens, 43.8% were positive for p-mTOR and a correlation between p-mTOR expression and smaller and lower-grade tumors was observed.	[124]
**Prostate**	Protein levels of mTOR are double in cancer tissue compared to normal and with a diffuse distribution, whereas p-mTOR localized in the cytoplasm, and presented a more focal expression (being also elevated in cancer and prostate intraepithelial neoplasia cells)	[125]
**Ovarian**	Expression of mTOR was up-regulated in PEO1TaxR (paclitaxel-resistant) ovarian cancer cells when compared with paclitaxel-sensitive PEO1 cells; and downregulated in SKOV-3TaxR (paclitaxel-resistant) cells when compared with the parental cellular counterparts.	[126]
	Expression of mTOR was increased in endometriosis and ovarian endometrioid adenocarcinoma patients compared to normal samples.	[127]
**Leukemia**	Expression of mTOR and p-mTOR was associated higher risk of paediatric acute lymphoblastic leukaemia (ALL) relapse.	[128]
	Expression of mTOR was upregulated in more than 50% of cases of ALL, both T- or B-ALL, and it was observed that the mean fold change of mTOR expression was higher in patients that did not respond to chemotherapy.	[53]
**Multiple Myeloma**	Expression of mTOR or p-mTOR was observed in 25.8% FFPE samples from MM patients and high mTOR and p-mTOR were associated with male gender and older patients.	[129]
**Sacral chordoma**	Expression of mTOR observed in 62.5% (25/40) cases of the sacral chordoma and it was associated with tumor invasion into the surrounding muscles.	[130]

Deregulation of mTOR signaling contributes to gastric cancer through several mechanisms, such as inhibition of apoptosis, induction of chemo-resistance phenotype, metastasis, epithelial to mesenchymal transition, and angiogenesis [131]. Additionally, some studies also demonstrated that elevated mTOR expression is indicative of the disease, as it is observed in gastric cancer samples in opposition to normal tissue/samples [115, 116, 132]. A similar behaviour occurs in prostate cancer, in which the levels of mTOR and 4EBP are high [125] and in esophageal squamous cancer, multinodular hepatocellular carcinoma and in ovarian endometrioid adenocarcinoma that present high expression of mTOR in opposition to normal tissues [118, 119, 127, 133]. These data might indicate that elevated mTOR expression might contribute to the pathogenesis of gastric, prostate, esophageal, liver, and ovarian tumours and that it might be useful as a **diagnostic biomarker**. In opposition to that, in paediatric acute lymphoblastic leukaemia mTOR expression was found to be more frequent at relapsed cases than at the first diagnosis of the disease [128].

In addition to be associated with disease onset, it seems that high mTOR expression assists **disease progression**, as it occurs in prostate cancer, in which high expression of mTOR is observed in both prostate intraepithelial neoplasia and cancer samples, being higher in cancer cells [125]. This seems to parallel the activation of mTOR pathway in prostate cancer, as the expression of activated mTOR (p-mTOR) is increased across samples of a progression cancer model (normal prostate tissue, proliferative inflammatory atrophy, prostatic intraepithelial neoplasia and cancer samples) [134]. In gastric cancer elevated mTOR expression seems to be associated with tumor differentiation, lymph node metastasis and clinical staging [116], tumor progression and poor survival [135, 136]. In these settings, elevated mTOR levels paralleled mTOR signaling activation. Similarly, in esophageal squamous cancer high expression of mTOR seems to be is associated with the occurrence of lymph node metastases, higher tumor grade and advanced TNM stage [119, 133]. The association of high mTOR expression and higher tumor grade also occurs in hepatocellular carcinoma[118]. Accordingly, elevated mTOR expression accompanies a more aggressive phenotype and might anticipate a poor **prognosis**. Indeed, Wu and colleagues demonstrated that overexpression of mTOR and mTOR hyperactivation were independent adverse prognostic factors for overall survival in esophageal tumors [119]. This data contrasted with a previous study that concluded that activation of mTOR was not related to patient survival [133]. Nevertheless, the former study addressed a larger sample [119] which might explain the differences in both studies. Indeed, other types of tumors parallel the association between high mTOR activation and shorter survival, as in pancreatic neuroendocrine cancer [123]. In this case, expression of mTOR correlates with mTOR activation, and is present in the majority of tumor samples [123]. In sacral chordoma, a locally aggressive malignant bone tumour, expression of mTOR was associated with tumor invasion into the surrounding muscles, suggesting a role for mTOR in local invasiveness [130]. For breast cancer, mTOR expression and activation seems to impact differently in different cancer subtypes. In luminal specimens, high expression seems to have a protective role and is associated with smaller and lower-grade tumors [124] whereas in triple negative breast cancer, mTOR signaling activation is seen in specimens with bigger size, lymph node metastasis, advanced stage and shorter overall survival [137]. Additionally, in a study addressing true interval cancers - tumours that appear after a negative screening mammogram and have a worse clinical behaviour- and screen-detected cancers, it was found that whereas all the former cases presented mTOR hyperactivation, only a third of the latter had it [138]. Similarly, in lung cancer, the profile of mTOR levels fluctuates between different cancer subtypes, with particular effects on the clinical behaviour of the tumour. In the case of non-small cell lung cancer (NSCLC), some authors have been suggesting that elevated mTOR levels could be used as a biomarker to predict the outcome of the disease. Nevertheless, a meta-analysis including results from ten studies demonstrated that there is no association between the levels of both total and activated mTOR and the prognosis of the disease[113]. This conclusion was obtained using both univariate analysis and multivariate analysis [113]. Nevertheless, other study demonstrated that in the EGFR-mutant NSCLC subtype, high mTOR expression was associated with shorter overall and progression-free survival in response to erlotinib, suggesting that inhibition of mTOR in those settings might be beneficial [114]. As pointed by the authors of the meta-analysis [113], it would be interesting to further explore the potential of mTOR expression as a prognostic biomarker in NSCLC as some methodological divergences in the analysed studies, namely the different cut-off points used to classify the samples as positive or negative for mTOR expression, could result in biased conclusions from the meta-analysis. Additionally, regarding to mTOR pathway, in typical carcinoid tumor (TC) and atypical carcinoid tumor (AC) the activation of mTOR signaling is observed in opposition to large-cell neuroendocrine carcinoma (LCNEC) and small-cell lung carcinoma (SCLC) [139]. Of note, TC and AC are less aggressive forms of bronchopulmonary neuroendocrine tumors, whereas SCLC and LCNEC are more aggressive, respectively[140]. Accordingly, in these subgroups of tumors, mTOR activation occur in less invasive phenotypes. Nevertheless, the authors found that in the more aggressive forms, LCNEC and SCLC, a positive association between mTOR signaling induction and tumor size was observed [139]. It would be interesting to determine whether mTOR expression accompanies mTOR signalling activation in those settings.

As for laryngeal carcinoma mTOR seems to potentiate a more aggressive evolution of the disease, and when expressed at high levels is associated with an increased rate of **disease recurrence** and shorter **disease-free survival** [120, 121]. Similarly, elevated mTOR levels are not related to differentiation or microvascular invasion in multinodular hepatocellular carcinoma (HCC), but are associated with higher disease recurrence after liver transplantation (LT) [118]. As LT is the therapeutic option with the superior 5-year survival rate, it is commonly chosen in cases of HCC and other liver diseases [141]. According to the data from this study, evaluation of mTOR expression could aid to predict whether LT will be a suitable strategy and/or to determine the utility to combine LT with mTOR inhibition in HCC patients. Similarly, in urothelial carcinoma, mTOR protein levels are elevated in patients with advanced stage of the disease and that do not respond to neoadjuvant **chemotherapy** [122]. In those cases, elevated expression of mTOR and p-mTOR is also observed in tumour microenvironment (peritumoral and normal stroma areas). Besides mTOR itself, it is observed that a worse phenotype of the disease is dictated by the upregulation of other genes from the mTOR pathway [122]. A study addressing mTOR expression in patients with B- and T- acute lymphoblastic leukaemia (ALL) revealed that patients with higher mTOR expression did not respond to chemotherapy, regardless of other prognostic factors [53]. Interestingly, in this study it was observed that children displayed almost two times higher expression of mTOR than adults [53]. These data indicate that elevated mTOR can be a useful biomarker to predict response to therapy, particularly in children. Nevertheless, one must note that this study evaluated mTOR mRNA and not protein levels and it is now known that mTOR undergoes post-transcriptional regulation [90]. In ovarian carcinoma cell lines, expression of mTOR has a cell-specific behavior, as it is up-regulated in PEO1TaxR (paclitaxel-resistant) ovarian cancer cells when compared to paclitaxel-sensitive PEO1 cells; and downregulated in SKOV-3TaxR (paclitaxel-resistant) cells comparing to the parental counterparts. Collectively, these data demonstrates that mTOR expression might be used as a biomarker to predict disease recurrence and/or response do therapy, and that the impact of mTOR overexpression must be interpreted according to the disease subtype.

Accordingly, mTOR overexpression seems to play a role in the pathogenesis of some conditions, both cancerous and non-cancerous thus presenting a potential value as a biomarker for **diagnosis** of gastric, prostate, esophageal, liver and ovarian cancer; **disease progression and phenotype** of prostate, gastric and esophageal cancer; **prognosis and invasive behavior** of esophageal, sacral, breast, lung and gastric cancer and **disease recurrence and response to therapy** in leukemia, laryngeal, liver, urothelial and ovarian cancer.

## Conclusion

The mTOR signalling is tightly controlled through several proteins and pathways that converge on this hub to connect extracellular environment and/or cellular needs with an adaptative cellular response. It is now becoming apparent that the regulation of the expression of mTOR itself adds another level of complexity. Although the studies about the regulation of mTOR expression are scarce, they provide evidence that mTOR transcription is increased to promote milk synthesis, through dynamic epigenetic modifications. Additionally, the use of an alternative and cap-independent mechanism of translation initiation both in normal and stressed conditions might explain how mTOR can be overexpressed and hyperactivated in a variety of conditions associated with a low-energy consumption state. Our group is establishing a mechanism to inhibit the cis-acting element present in the mTOR 5’UTR that assists the recruitment of the ribosome to this transcript, to reduce mTOR overexpression.

Besides these advances, it would be interesting to further address how is mTOR expression regulated. The epigenetic modifications that occur during milk synthesis in mammary cells are a universal event for *mTOR* transcriptional regulation? Are there any other epigenetic marks regulating mTOR? Do *mTOR* have other specific elements, either acting in cis or trans, to regulate the production of *mTOR* mRNA? In terms of mRNA stability, how do occur the binding of LARP1 to mTOR mRNA? Are there other proteins/elements/processes regulating mTOR mRNA stability?

By converging so many signals and pathways, it comes without surprise that mTOR signalling is deregulated in a huge fraction of the human diseases. Furthermore, mTOR overexpression seems to play a role in the pathogenesis of some conditions or at least signalize the onset and/or the progression of some non-cancerous diseases, in some of which it is associated with a poor outcome. As for cancer, data indicate that mTOR overexpression has a potential value as a biomarker for diagnosis of gastric, prostate, esophageal, liver and ovarian cancer; disease progression and phenotype of prostate, gastric and esophageal cancer; prognosis and invasive behavior of esophageal, sacral, breast, lung and gastric cancer and disease recurrence and response to therapy in leukemia, laryngeal, liver, urothelial and ovarian cancer. Accordingly, reduction of mTOR expression might constitute the goal of the next generation of drugs targeting mTOR. Indeed, it has been demonstrated that compounds that suppress cell proliferation and induce apoptosis do so by reducing mTOR expression levels [142]. A compound specifically targeting mTOR with a robust decrease of its expression might be the key to counteract mTOR signalling.

## Data Availability

Not applicable.
